# Effectiveness of Inactivated COVID-19 Vaccines against COVID-19 Caused by the SARS-CoV-2 Delta and Omicron Variants: A Retrospective Cohort Study

**DOI:** 10.3390/vaccines10101753

**Published:** 2022-10-19

**Authors:** Qiaoli Hua, Danwen Zheng, Bo Yu, Xinghua Tan, Qiumin Chen, Longde Wang, Jing Zhang, Yuntao Liu, Heng Weng, Yihang Cai, Xiaohua Xu, Bing Feng, Guangjuan Zheng, Banghan Ding, Jianwen Guo, Zhongde Zhang

**Affiliations:** 1 The Second Clinical College, Guangzhou University of Chinese Medicine, Guangzhou 510006, China; 2 Department of Emergency, the Second Affiliated Hospital of Guangzhou University of Chinese Medicine, Guangzhou 510120, China; 3 Guangdong Provincial Key Laboratory of Research on Emergency in Traditional Chinese Medicine, Guangzhou 510120, China; 4 Department of General Surgery, The No.2 People’s Hospital of Lanzhou, Lanzhou 730030, China; 5 Department of Traditional Chinese Medicine, Guangzhou Eighth People’s Hospital, Guangzhou Medical University, Guangzhou 510060, China; 6 Department of Traditional Chinese Medicine, Xinglin District, The First Affiliated Hospital of Xiamen University, Xiamen 361022, China; 7 Affiliated Hospital of Gansu University of Traditional Chinese Medicine, Lanzhou 730020, China; 8 Department of Respiratory and Critical Care Medicine, Xiamen Hospital of Traditional Chinese Medicine, Xiamen 361000, China; 9 State Key Laboratory of Dampness Syndrome of Chinese Medicine, Guangzhou 510120, China; 10 Department of Neurology, The Second Affiliated Hospital of Guangzhou University of Chinese Medicine, Guangzhou 510120, China; 11 Department of Pharmacology of Traditional Chinese Medicine, the Second Affiliated Hospital of Guangzhou University of Chinese Medicine, Guangzhou 510120, China

**Keywords:** coronavirus disease 2019, the Delta variant, the Omicron (BA.2.38) variant, inactivated COVID-19 vaccine, a retrospective cohort study

## Abstract

Background: Real-world evidence on the effectiveness of inactivated vaccines against the Delta and Omicron (BA.2.38) variants remains scarce. Methods: A retrospective cohort study was conducted to estimate the adjusted vaccine effectiveness (aVE) of one, two, and three doses of inactivated vaccines in attenuating pneumonia, severe COVID-19, and the duration of viral shedding in Delta and Omicron cases using modified Poisson and linear regression as appropriate. Results: A total of 561 COVID-19 cases were included (59.2% Delta and 40.8% Omicron). In total, 56.4% (184) of Delta and 12.0% (27) of Omicron cases had COVID-19 pneumonia. In the two-dose vaccinated population, 1.4% of Delta and 89.1% of Omicron cases were vaccinated for more than 6 months. In Delta cases, the two-dose aVE was 52% (95% confidence interval, 39–63%) against pneumonia and 61% (15%, 82%) against severe disease. Two-dose vaccination reduced the duration of viral shedding in Delta cases, but not in booster-vaccinated Omicron cases. In Omicron cases, three-dose aVE was 68% (18%, 88%) effective against pneumonia, while two-dose vaccination was insufficient for Omicron. E-values were calculated, and the E-values confirmed the robustness of our findings. Conclusions: In Delta cases, two-dose vaccination within 6 months reduced pneumonia, disease severity, and the duration of viral shedding. Booster vaccination provided a high level of protection against pneumonia with Omicron and should be prioritized.

## 1. Introduction

The coronavirus disease 2019 (COVID-19) pandemic has caused great public health concerns worldwide. COVID-19 vaccines play an indispensable role in curtailing the pandemic over the world. Using whole-virus inactivation technology, two inactivated vaccines, the Beijing Institute of Biological Products Co., Ltd., Beijing, China (BIBP) vaccine (developed by China National Biotec Group, Sinopharm) and the CoronaVac vaccine (Sinovac Biotech Ltd., China) have been used in more than 80 countries [1,2,3] and were listed for emergency use by WHO. By 6 September 2022, more than 3.4 billion doses have been administrated in China [4], most of which were inactivated COVID-19 vaccines.

According to randomized, double-blind, phase-three trials, the BIBP vaccine has an efficacy of 78.1% against symptomatic infection and 100% against severe disease [5]. The CoronaVac vaccine has an efficacy ranging from 51% to 84% against symptomatic infection and 85–100% against hospitalization [6,7,8]. Similar vaccine effectiveness (VE) against severe COVID-19 associated with the Delta variant was also reported [2,5,9,10,11]. As the continuing mutation of SARS-CoV-2 [12], the Omicron (B.1.1.529) variant, which is highly transmissible with potential immune escape, raised concerns about vaccine effectiveness. Studies reported decreased neutralizing activity against Omicron compared to wild-type SARS-CoV-2 for inactivated vaccines [13,14]. Additionally, waning protection of two-dose CoronaVac against Omicron [11,15] has been reported. Another study in Hongkong suggested that the VE of three-dose CoronaVac against severe or fatal COVID-19 (Omicron, BA.2) was high [15]. However, real-world evidence on inactivated VE against the more transmissible Omicron (BA.2.38) variants remains scarce.

Different from some other countries, no large-scale local outbreak emerged after the first epidemic wave in 2020 due to the effective implementation of the “zero-infection strategy” in China. Thus, the immunity of the Chinese population is almost entirely from COVID-19 vaccines, rather than hybrid immunity from previous infections and vaccination. In May and September of 2021, the B.1.617.2 (Delta) variant emerged in Guangzhou and Xiamen, China, respectively. Then, in 2022, the Omicron (B.1.1.529) variant rapidly became the dominant variant in China. A local Omicron (BA.2.38) outbreak occurred in Lanzhou, China in July 2022. These outbreaks provide a unique opportunity to monitor VE against Delta and Omicron (lineage BA.2.38). We conducted a retrospective cohort study to evaluate the effectiveness of one-dose, two-dose, and three-dose inactivated vaccines in reducing the proportion of pneumonia, severe disease, and the duration of viral shedding.

## 2. Methods

### 2.1. Study Design and Participants

A retrospective cohort study involving 828 cases was conducted in three COVID-19 designated hospitals in China, with 166 from Eighth People’s Hospital of Guangzhou (the Delta variant) from 21 May to 18 June 2021, 243 from the First Affiliated Hospital of Xiamen University (the Delta variant) from 10 September to 3 October 2021, and 419 from the Second People’s Hospital of Lanzhou City (the Omicron variant, BA.2.38) from 11 July to 26 July 2022. These cases were all consecutive cases with real-time fluorescence quantitative PCR (RT-PCR)-confirmed COVID-19 and were local cases from the same transmission chain in each city. As COVID-19 vaccines were only provided to adults until July 2021 according to the nation’s policy, those younger than 18 years old were excluded. The following cases were also excluded: those who were pregnant; those who were vaccinated with non-inactivated vaccines; and those with no information regarding clinical outcomes or vaccination status. The China government recommended a booster vaccination (3 doses) in October 2021. As a result, only 4 Delta cases received booster shots and 6 Omicron cases were partially vaccinated (1 dose), and these cases were also excluded. All cases infected with SARA-CoV-2 in our study were hospitalized regardless of disease severity.

This study was approved by the Ethical Committee of Guangdong Provincial Hospital of Chinese Medicine (No. ZE2021-114-01) and was registered on Chictr.org.cn (ChiCTR2200060714). The informed consent was waived due to the study’s observational nature.

### 2.2. Diagnostic Criteria and Definitions

A confirmed case of COVID-19 was defined based on the national diagnosis and treatment protocol (8th version in 2021 and 9th in 2022) for COVID-19 in China [16,17]. Confirmed cases were classified as mild, moderate, severe, and critical cases [16,17]. Severe COVID-19 cases were defined as cases with a respiratory rate ≥ 30/min, a resting oxygen saturation ≤ 93%, and an oxygenation index ≤ 300 mmHg, or pulmonary lesion progression greater than 50% within 24–48 h. Critical COVID-19 cases were cases that met any of the following criteria: respiratory failure requiring mechanical ventilation, shock, or organ failure requiring admission to the ICU. In further analysis, cases who progressed to severe and critical disease were combined as severe cases and the other were combined as non-severe cases. Pneumonia was diagnosed by clinical symptoms and chest CT imaging, such as ground-glass opacification with or without consolidative abnormalities

### 2.3. Vaccination Status

As two weeks were needed to fully form an immune response to vaccination, cases were deemed as unvaccinated if they received only 1 dose, but the time interval from vaccination to clinical diagnosis was less than 14 days. Finally, cases were divided into three groups: unvaccinated (0 doses, comprising those <14 days after the first dose), partial vaccination (1 dose, comprising those <14 days after the second dose), full vaccination (2 doses, comprising those <14 days after the third dose), and booster vaccination (3 doses).

### 2.4. Information Collection

All data were extracted from electrical medical records in the hospitals. The following information was collected: demographic characteristics (sex, age, comorbidities), vaccination status (doses of vaccination, date of vaccination, and manufacturer), laboratory findings (white blood cell counts, neutrophil counts, lymphocyte counts, D-dimer, C-reactive protein, and IL-6), the cycle threshold value (Ct-value of ORF1ab and N targets, a proxy for viral load), IgG and IgM antibody tilter (RBD-specific), and the duration of viral shedding (defined as the time from the first positive SARS-CoV-2 RNA test to a successive negative test). Vaccination status was obtained by doctors from the health code on the patient’s mobile phone.

### 2.5. Statistical Analysis

Data were presented as the mean (S.D.) or median (IQR) for normally and nonnormally distributed data, respectively. Comparisons of continuous variables between the groups were performed by the *t*-test, ANOVA, the Mann–Whitney U test, and the Kruskal–Wallis test as appropriate. Categorical variables were analyzed by the chi-square test and Fisher’s exact test. A value of *p* < 0.05 was considered statistically significant.

The primary outcome was the proportion of pneumonia. Secondary outcomes included the proportion of severe disease and the duration of viral shedding. Modified Poisson regression [18] was used to evaluate the relative risk (RR) of vaccination on pneumonia and disease progression without and with adjusted for age, gender, and whether the case had comorbidities (i.e., chronic kidney disease, cancer, chronic respiratory disease, chronic liver disease, hyperlipidemia, immune-compromised status, chronic cardiovascular disease, cerebrovascular disease, diabetes, and hypertension). Modified Poisson regression with robust estimation of variance for binary non-replicated outcomes was used to estimate RR when the assumption of rare diseases was violated. The Poisson distribution’s mean–variance relationship may not be appropriate for binary outcomes, so we used robust estimates of variance. The adjusted risk ratios (aRRs) of each outcome were calculated in reference to the unvaccinated group and the adjusted vaccine effectiveness(aVE) was then calculated as 100% × (1 − aRR). For the protective effects of vaccines, we used aRRs rather than odds ratios (ORs) to calculate aVE because ORs consistently underestimated RR and thus led to an overestimation of VE.

Linear regression models were used to evaluate the association between vaccination and duration of viral shedding without and with adjusted for age, gender, and whether the case had comorbidities and cycle threshold (Ct) values at admission.

### 2.6. Sensitivity Analyses

We further explore the association of disease progression with vaccination status in different subgroups. These groups were divided according to older age (≥60 years), whether they had comorbidities, and gender. Furthermore, to explain the effect of vaccination status on different outcomes, we used E-value [19] to evaluate the potential effects of unmeasured confounding. The E-value quantifies the required magnitude of an unmeasured confounder that could negate the observed association between exposure (for example, vaccination) and the outcomes (for example, pneumonia). All analyses were performed using R (v.3.3.2, R Foundation for Statistical Computing, Vienna, Austria; http://www.R-project.org, accessed on 1 January 2021) and the Free Statistics analysis platform (v.1.7).

## 3. Results

### 3.1. Study Population

**Figure 1:**
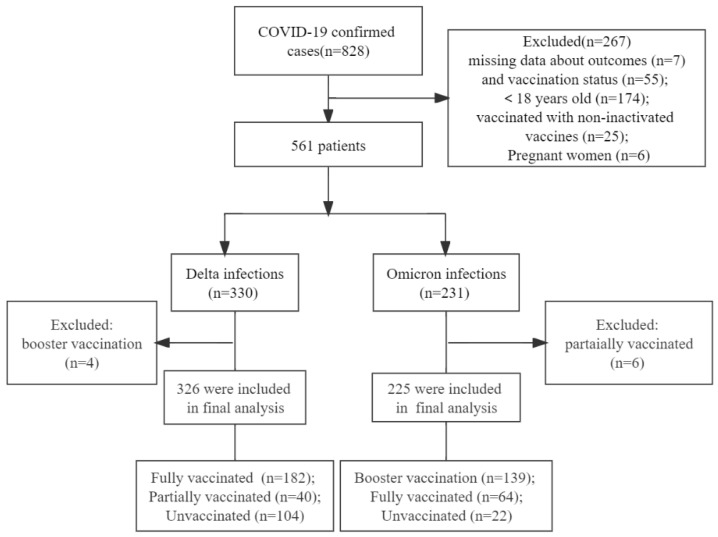
Study flow chart.

Among 828 cases infected with the Delta and Omicron variant, 62 (7.5%) missed information related to outcomes or vaccination status, 174 (21.0%) were younger than 18 years old, 25 (3.0%) were vaccinated with non-inactivated vaccines, 6 (0.7%) were pregnant women, 4 (0.9%) were booster-vaccinated in Delta cases, and 6 (0.7%) were partially vaccinated in Omicron cases, so these cases were subsequently excluded. Consequently, 326 Delta and 225 Omicron cases were included (Figure 1). All included patients were vaccinated with BIBP or CoronaVac. No cases had been previously infected with SARS-CoV-2.

### 3.2. Characteristics of Participants

The demographic and clinical characteristics of the cases are shown in Table 1. Of the 551 cases, 176 (54.0%) Delta cases and 137 (60.9%) Omicron cases were female. In total, 163 (29.6%) had comorbidities. The median age was 44.0 years. Among 326 Delta cases, 182 (55.8%) were fully vaccinated, 40 (12.3%) were partially vaccinated, and 104 (31.9%) were unvaccinated. As the process of immunization campaigns in China, by July 2022, most individuals were fully and booster-vaccinated. Therefore, in Omicron cases that occurred in July 2022, 139 (61.8%) were booster-vaccinated, 64 (28.4%) were fully vaccinated, and 22 (9.8%) were unvaccinated. Furthermore, 56.4% of Delta and 12% of Omicron cases progressed to pneumonia. Omicron cases had a shorter duration of viral shedding (11.0 days in Omicron vs. 16.5 days in Delta), and a lower proportion of fever (42.7% in Omicron vs. 70.2% in Delta). No cases progressed to severe COVID-19 in Omicron infection.

### 3.3. Antibodies and Viral Loads among Different Groups

As shown in Table 2, among Delta and Omicron cases, with increasing doses of the vaccines, the IgG titers within 48 h from admission increased (*p* < 0.001). In terms of the maximum IgG titers, the same trend was observed among Omicron cases. In Delta and Omicron cases, no significant differences between vaccination status, in terms of IgM antibody titers, Ct values within 48 h from admission, or the lowest Ct values, were found. Table S1 describes serum inflammation indicators in Delta and Omicron cases. As vaccination doses increased, lymphocyte counts increased and IL-6 levels decreased in Delta cases. However, Omicron cases showed a decrease in lymphocyte counts and an increase in neutrophil counts and C-reactive protein.

### 3.4. Outcomes among Different Groups

**Figure 2:**
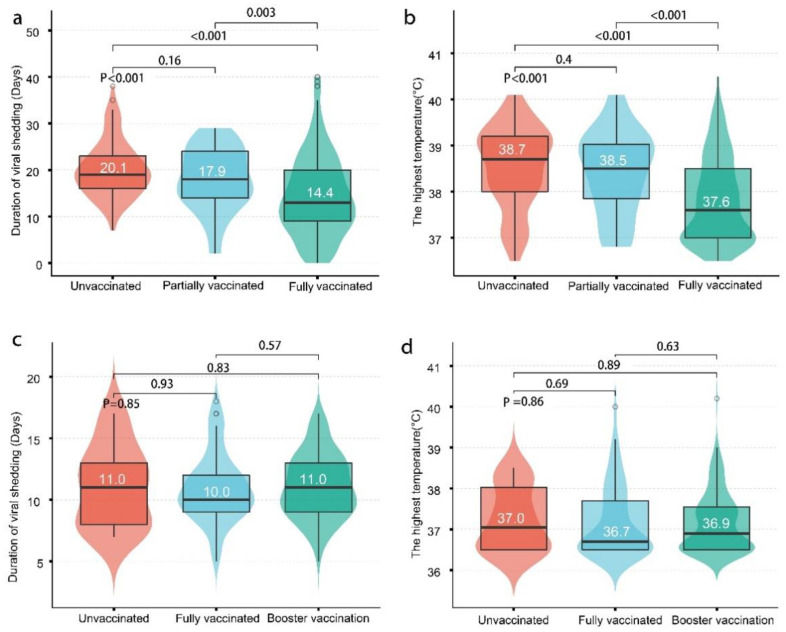
The duration of viral shedding and the highest temperature after Delta (**a**,**b**) and Omicron (**c**,**d**) infections. (**a**) The duration of viral shedding and (**b**) the highest temperature in Delta cases. As the dose of vaccines increased, the duration of viral shedding and the highest temperature decreased in Delta cases. (**c**) The duration of viral shedding and (**d**) the highest temperature in Omicron cases. No differences were found between different vaccination groups in the highest temperature or duration of viral shedding in Omicron cases. Boxes depict the interquartile range (IQR) and contain the median (black line) with whiskers spanning 1.5 times the IQR. The shaded area reflects the data distribution.

Table 3 and Figure 2 summarize the outcomes and intervals from the last shot to symptom onset. In fully vaccinated cases, the proportion of less than 6 months between vaccination and symptom onset was 98.4% in Delta and only 10.9% in Omicron cases. In Delta and Omicron cases, the proportion of pneumonia decreased as the dose of vaccines increased (86.5% in the unvaccinated group, 67.5% in the partially vaccinated group, and 36.8% in the fully vaccinated group; *p* < 0.001 for Delta) (31.8% in the unvaccinated group, 15.6% in the partially vaccinated (one-dose) group, and 7.2% in the fully vaccinated (two-dose) group; *p* = 0.005 for Omicron). In Delta cases, a similar trend was found in the proportion of severe COVID-19 (32.7% in unvaccinated, 12.5% in one-dose group, and 5.5% in the two-dose group; *p* < 0.001), the highest temperature (Figure 2a), and the duration of viral shedding (Figure 2b) (20.1 ± 6.0 days in the unvaccinated group, 17.9 ± 6.8 days in the one-dose group, and 14.4 ± 8.7 days in the two-dose group) (Table 2). In the Omicron cases, no cases developed severe COVID-19, and no differences were found between different vaccinated groups in terms of the duration of viral shedding (Figure 2c) or the highest temperature (Figure 2d).

### 3.5. Vaccine Effectiveness

Table 4 shows the adjusted VE (aVE) against different outcomes. In Delta cases, the two-dose aVE was 52% (95% CI, 39–63%) against pneumonia and 61% (95% CI, 15–82%) against the severe disease after adjustment for age, whether they had comorbidities and gender. The duration of viral shedding was 4.68 days less (95% CI, −6.89 to −2.46 days) in the two-dose group compared with the unvaccinated group with Delta. On the other hand, partial vaccination was not statistically associated with these outcomes. In Omicron cases, three-dose aVE was 68% (95% CI, 18–88%) against pneumonia, booster vaccination was not associated with a reduced duration of viral shedding, and full vaccination was not associated with any outcomes.

### 3.6. Sensitivity Analysis

As shown in Tables S2 and S3, we performed a stratified analysis to explore the VE of vaccination against pneumonia in different subgroups. No statistically significant difference was seen in two-dose or three-dose aVE against pneumonia in different subgroups. There was a trend toward decreased VE among Omicron cases with comorbidities.

Furthermore, we calculated the E-value to evaluate the potential effect of an unmeasured confounding on different outcomes. The E-value ranged from 2.647 to 4.567 for two-dose aVE against pneumonia, severe COVID-19, and the duration of viral shedding in Delta cases, and was 5.702 for three-dose aVE against pneumonia with Omicron, indicating that a strong concurrent confounder is required to change the observed aVE (Table S4).

## 4. Discussion

We assessed the effectiveness of two prevailing inactivated vaccines (BIBP and Conavac) against pneumonia, disease progression, and shortening the duration of viral shedding in Delta and Omicron cases in a real-world setting in China. In total, 56.4% (184) of Delta and 12.0% (27) of Omicron cases had COVID-19 pneumonia. In cases infected with the Delta variant, two-dose aVEs were 52% (95% CI, 39–63%) against pneumonia, 61% (95% CI, 15–82%) against severe COVID-19, and two-dose vaccination was statistically associated with a 4.68-day reduction in viral shedding. However, in Omicron cases, two-dose and three-dose vaccination did not result in a shorter duration of viral shedding. The aVE of three-dose vaccination was 68% (95% CI, 18–88%) against pneumonia with Omicron. By contrast, two-dose vaccination (89.1% were more than 6 months from second shot to infection) was insufficient for Omicron, and one-dose vaccination was insufficient for Delta.

The risk of pneumonia, severe COVID-19, and fever with Omicron were significantly lower than with Delta, which is consistent with previous reports indicating that Omicron causes less severe COVID-19 than Delta [20,21]. Our study provided evidence for the protective effect of two-dose inactivated vaccines against Delta and booster vaccination against the Omicron variant. Similar VEs against severe COVID-19 caused by the Delta variant were reported in other studies [2,5,9,10,11]. For example, two real-world studies estimated that the aVEs of inactivated vaccines were 100% (95% CI, 98.4% to 100.0%) [22], 88% (95% CI, 55% to 98%) [10], and 83% [11] against severe COVID-19. Different from these studies, we found that full vaccination was independently associated with a reduced duration of viral shedding in Delta infection. The viral clearance may be promoted by vaccine-induced immune because fully vaccinated Delta cases had a greater IgG-48h level, which was supported by another study [23]. However, in Omicron cases, three-dose VE was not statistically significant in reducing the duration of viral shedding, although the IgG-48h level and the maximum IgG level were higher than the unvaccinated individuals, reflecting the stronger immune escape and reduced VE in Omicron cases. The decreased VE against Omicron is also found in other vaccines, such as mRNA vaccines [24,25].

Although two-dose VE against pneumonia with Delta was high, two-dose VE against pneumonia with Omicron is inadequate, which was inconsistent with results from another study [20]. The inconsistency may be primarily due to the waning immunity with time since 89.1% of two-dose-vaccinated Omicron cases were more than 6 months from second shot to infection. Booster immunization with inactivated vaccines provided a high level of protection against pneumonia (aVE = 68%) with Omicron (BA.2.38), which was consistent with a study in Hong Kong [15]. These findings implied the importance of booster vaccination in persons >6 months after the second dose. Subgroup analyses indicated a trend toward decreased three-dose VE among Omicron cases with comorbidities. Since individuals with comorbidities were at higher risk of disease progression [26], monitoring VE against Omicron is significant to inform future vaccination strategies in these high-risk persons.

The attenuation effects of vaccines are biologically plausible based on similar phenomena observed in other vaccine studies [27,28,29,30]. The effects may be due to the recall of an immune memory response, reduced viral replication, and the accelerated eradication of virus-infected cells [27,31]. An in vivo study has demonstrated that prototypic inactivated vaccines reduced viral load in upper respiratory tract swabs and pulmonary tissues, and improved lung pathology in vaccinated rhesus monkeys which were infected by the Alpha, Beta, Delta, and Omicron variants [14].

No significant difference was found in SARS-CoV-2 viral loads among unvaccinated, one-dose, two-dose, and three-dose vaccination groups in Delta and Omicron infections. Similar results were found in other studies associated with inactivated vaccines [15,21]. By contrast, Li et al. observed viral loads decreased 1.9–3.4-fold in children who were fully vaccinated [32]. The notion of whether inactivated vaccines decreased viral loads needs to be further explored.

The study has several limitations. First, in multivariable regression analyses, despite the known confounding factors, such as age, gender, and comorbidities, which were adjusted, residual unmeasured confounders may affect the reliability of the analyses, but the E-value (two-dose vaccination in Delta and three-dose against pneumonia in Omicron cases) indicated the aVEs were robust unless a strong unmeasured confounder existed simultaneously to change the observed aVEs. Second, as neutralizing antibody levels elicited by COVID-19 vaccines waned substantially over time [33,34], 89.1% of two-dose-vaccinated and 47.5% of three-dose-vaccinated Omicron cases were more than 6 months from the last shot to infection. The prolonged interval may underestimate the VE. Third, the sample size was relatively small, which was reflected by the wide CIs. Fourth, we did not evaluate three-dose VE in Delta infections because only four cases were booster-vaccinated in Delta infections. Fifth, it was difficult to compare two-dose VE in Delta and Omicron infections since the time interval between the last vaccination shot to infection was different in the two groups.

Despite these limitations, cases in our study were not previously infected with SARS-CoV-2; thus, the pure vaccine-induced immunity could be estimated, which may provide insights into the inactivated VE against Delta and Omicron variants in a real-world setting. The findings suggested that continuing the mass booster immunization program was still critically important for the whole population against Omicron.

## 5. Conclusions

In Delta cases, full vaccination of the inactivated SARS-CoV-2 vaccines within 6 months provided strong protection against pneumonia and severe disease, and shortened the duration of viral shedding. In Omicron cases, booster vaccination offered a high level of protection against pneumonia, but full vaccination over 6 months could not. Booster immunization was still critically important as the waning immunity over time.

## Figures and Tables

**Table 1:** Demographic and clinical characteristics of cases infected with Delta and Omicron variants.

Variables	Total (*n* = 551)	Delta Infections (*n* = 326)	Omicron Infections (*n* = 225)	*p*
Gender, *n* (%)				0.108
Male	238 (43.2)	150 (46.0)	88 (39.1)	
Female	313 (56.8)	176 (54.0)	137 (60.9)	
Age, (years old)	44.0 (35.0, 56.0)	45.0 (36.0, 58.0)	42.0 (33.0, 55.0)	0.029
Age < 60, *n* (%)	451 (81.9)	258 (79.1)	193 (85.8)	0.047
Age ≥ 60, *n* (%)	100 (18.1)	68 (20.9)	32 (14.2)	
Comorbidities, *n* (%)	163 (29.6)	135 (41.4)	28 (12.4)	<0.001
Hypertension, *n* (%)	70 (12.7)	53 (16.3)	17 (7.6)	0.003
Diabetes, *n* (%)	23 (4.2)	18 (5.5)	5 (2.2)	0.057
Chronic lung disease, *n* (%)	38 (6.9)	37 (11.3)	1 (0.4)	<0.001
Uncured cancer, *n* (%)	8 (1.5)	5 (1.5)	3 (1.3)	1.000 ^a^
Cardiovascular disease, *n* (%)	16 (2.9)	11 (3.4)	5 (2.2)	0.429
Cerebrovascular disease, *n* (%)	5 (0.9)	5 (1.5)	0 (0.0)	0.083 ^a^
Chronic liver disease, *n* (%)	20 (3.6)	20 (6.1)	0 (0.0)	<0.001
Chronic kidney disease, *n* (%)	6 (1.1)	6 (1.8)	0 (0.0)	0.086
Immune compromised ^b^, *n* (%)	3 (0.5)	3 (0.9)	0 (0.0)	0.274 ^a^
Vaccination status, *n* (%)				<0.001
Unvaccinated (0 dose)	126 (22.9)	104 (31.9)	22 (9.8)	
Partially vaccinated (1 dose)	40 (7.3)	40 (12.3)	0 (0.0)	
Fully vaccinated (2 doses)	246 (44.6)	182 (55.8)	64 (28.4)	
Booster-vaccinated (3 doses)	139 (25.2)	0 (0.0)	139 (61.8)	
Median days from last vaccination shot to symptom onset, (IQR)	93.5 (42.0, 188.5)	52.0 (30.2, 92.0)	197.0 (143.5, 276.0)	<0.001
Pneumonia, *n* (%)	211 (38.3)	184 (56.4)	27 (12.0)	<0.001
Clinical severity, *n* (%)				<0.001
Asymptomatic	71 (12.9)	10 (3.1)	61 (27.1)	
Non-severe ^c^	431 (78.2)	267 (81.9)	164 (72.9)	
Severe ^d^	49 (8.9)	49 (15.0)	0 (0.0)	
The duration of viral shedding, (days, IQR)	13.0 (10.0, 18.0)	16.5 (11.0, 22.0)	11.0 (9.0, 13.0)	<0.001
The highest temperature, *n* (%)				<0.001
<37.3 °C	226 (41.0)	97 (29.8)	129 (57.3)	
37.3–38 °C	128 (23.2)	66 (20.2)	62 (27.6)	
38.1–39 °C	130 (23.6)	100 (30.7)	30 (13.3)	
≥39.1 °C	67 (12.2)	63 (19.3)	4 (1.8)	

^a^*p* value was estimated by Fisher’s test for categorical variables; ^b^ immune compromised included patients after transplantation, receiving immune suppressive medications, or HIV infection; ^c^ bon-severe cases consist of mild and moderate patients; ^d^ severe cases consist of severe and critical patients. The paratheses for quantitative variables refer to the interquartile range (IQR). Ordinally scaled parameters (such as vaccination status, clinical severity, or temperature) were tested by the Mann–Whitney U test.

**Table 2:** Antibody titers and viral loads after the Delta and Omicron infections stratified by vaccination status.

Variables	Delta Variant				Omicron Variant			
	Unvaccinated (*n* = 104)	Partially Vaccinated (*n* = 40)	Fully Vaccinated (*n* = 182)	*p*	Unvaccinated (*n* = 22)	Fully Vaccinated (*n* = 64)	Booster Vaccination (*n* = 139)	*p*
IgG-48h ^a^	0.0 (0.0, 0.1)	0.6 (0.1, 3.3)	15.3 (3.5, 52.4)	<0.001	0.1 (0.1, 0.4)	1.3 (0.4, 4.9)	7.8 (3.1, 18.9)	<0.001
IgM-48h ^a^	0.0 (0.0, 0.1)	0.1 (0.1, 0.2)	0.4 (0.1, 1.6)	<0.001	0.1 (0.1, 0.3)	0.3 (0.1, 0.7)	0.2 (0.1, 0.6)	0.082
IgG max ^b^	10.4 (2.7, 25.7)	19.0 (3.4, 71.4)	7.4 (2.4, 25.2)	0.292	0.2 (0.1, 0.5)	2.8 (0.8, 52.2)	19.9 (5.9, 82.2)	<0.001
IgM max	168.3 (15.8, 448.4)	169.0 (39.1, 344.6)	330.8 (254.4, 386.3)	0.295	0.1 (0.1, 0.3)	0.4 (0.1, 1.1)	0.3 (0.2, 0.9)	0.068
Viral load (Ct value ^c^, within 48 h of admission)								
ORF1ab target	26.0 ± 6.1	26.9 ± 6.9	23.8 ± 6.0	0.387	26.0 (22.0, 33.0)	29.0 (24.5, 32.0)	27.0 (22.0, 31.0)	0.382
*n* target	23.7 (19.6, 29.9)	26.3 (19.6, 30.1)	22.4 (20.0, 26.1)	0.671	28.0 (23.0, 33.0)	29.0 (26.0, 32.0)	28.0 (22.0, 31.0)	0.150
Viral load (lowest Ct value)								
ORF1ab target	21.9 (19.3, 25.1)	20.7 (18.2, 24.3)	19.5 (18.4, 22.6)	0.239	26.0 (22.0, 32.0)	27.0 (23.0, 30.0)	26.0 (21.0, 30.0)	0.179
*n* target	20.5 (17.9, 23.7)	19.0 (17.1, 22.0)	18.1 (16.5, 20.9)	0.203	27.0 (23.0, 31.0)	27.0 (23.2, 31.0)	26.0 (22.0, 30.0)	0.092

^a^ IgG and IgM (RBD-specific) were obtained within 48 h from admission. ^b^ IgG was the maximum IgG titers from admission to discharge. ^c^ Ct value, cycle threshold value, which is a proxy for viral load. A lower Ct value indicated a higher viral load. Missing data for IgM and IgG (Delta variant), *n* = 193 (59.2%, all the missed data were from Xiamen); viral load (Delta variant), *n* = 193 (59.2%, all the missed data were from Xiamen). IgM-48h and IgG-48h (Omicron variant): *n* = 81 (36.0%); IgM max and IgG max (Omicron variant): *n* = 57 (25.3%). The Ct values for the ORF1ab target in Delta cases were given as mean ± SD. The other variables were given as median, IQR.

**Table 3:** Outcomes and interval from last shot to symptom onset in Delta and Omicron infections stratified by vaccination status.

Variables	Delta Variant				Omicron Variant			
	Unvaccinated (*n* = 104)	Partially Vaccinated (*n* = 40)	Fully Vaccinated (*n* = 182)	*p*	Unvaccinated (*n* = 22)	Fully Vaccinated (*n* = 64)	Booster-Vaccinated (*n* = 139)	*p*
Pneumonia	90 (86.5)	27 (67.5)	67 (36.8)	<0.001	7 (31.8)	10 (15.6)	10 (7.2)	0.005
Severe COVID-19 ^a^	34 (32.7)	5 (12.5)	10 (5.5)	<0.001	0 (0.0)	0 (0.0)	0 (0.0)	-
Duration of viral shedding	20.1 ± 6.0	17.9 ± 6.8	14.4 ± 8.7	<0.001	11.0 (8.0, 13.0)	10.0 (9.0, 12.0)	11.0 (9.0, 13.0)	0.847
The highest temperature	38.7 (38.0, 39.2)	38.5 (37.9, 39.0)	37.6 (37.0, 38.5)	<0.001	37.0 (36.2, 38.0)	36.7 (36.4, 37.7)	36.9 (36.4, 37.5)	0.96
The interval from last vaccination shot to symptom onset	-	29.0 (18.8, 43.5)	61.0 (35.0, 97.0)	<0.001	-	320.0 (285.0, 376.0)	182.6 (130.9, 199.0)	<0.001
≤180 days, *n* (%)	-	40 (100)	179 (98.4)	<0.001	-	7 (10.9)	73 (52.5)	<0.001
>180 days, *n* (%)	-	0 (0.0)	3 (1.4)		-	57 (89.1)	66 (47.5)	

^a^ consists of severe and critical cases.

**Table 4:** VE in cases infected with Delta and Omicron variants.

a. VE against Pneumonia and Disease Progression in Delta and Omicron Cases										
Outcomes	Delta Infection					Omicron Infection				
	Events/ Cases (%)	Unadjusted VE (95% CI)	*p* Value	Adjusted VE (95% CI)^a^	*p* Value	Events/ Cases (%)	Unadjusted VE (95% CI)	*p* Value	Adjusted VE (95% CI)^a^	*p* Value
Pneumonia										
Unvaccinated	90/104 (86.5)	Reference		Reference		7/22 (31.8)	Reference		Reference	
Partially vaccinated	27/40 (67.5)	22% (−4%, 41%)	0.089	12% (−21%, 35%)	0.442	-	-		-	
Fully vaccinated	67/182 (36.8)	57% (48%, 66%)	<0.001	52% (39%, 63%)	<0.001	10/64 (15.6)	51% (−22%, 80%)	0.128	32% (−70%, 73%)	0.408
Booster-vaccinated	-	-		-		10/139 (7.2)	77% (44%, 91%)	0.002	68% (18%, 88%)	0.019
Severe or critical										
Unvaccinated	34/104 (32.7)	Reference		Reference		0/22	Reference		Reference	
Partially vaccinated	5/40 (12.5)	62% (9%, 84%)	0.031	8% (−141%,65%)	0.863	-	-		-	
Fully vaccinated	10/182 (5.5)	83% (68%, 91%)	<0.001	61% (15%, 82%)	0.018	0/64	-		-	
Booster-vaccinated	-	-		-		0/139	-		-	
**b. VE in Shorting Duration of Viral Shedding in Delta and Omicron Cases**										
**Vaccination** **Status**	**Delta Infection**					**Omicron Infection**				
	**Duration of** **Viral Shedding (days)**	**Unadjusted β (95% CI)**	***p* Value**	**Adjusted β** **(95% CI) ^a^**	***p* Value**	**Duration of** **Viral Shedding (days)**	**Unadjusted β (95% CI)**	***p* Value**	**Adjusted β (95% CI) ^b^**	***p* Value**
Unvaccinated	20.1 ± 6.0	Reference		Reference		11.1 ± 3.3	Reference		Reference	
Partially vaccinated	17.9 ± 6.8	−2.21 (−5.03, 0.62)	0.127	−1.18 (−4.21~1.84)	0.444	-	-		-	
Fully vaccinated	14.4 ± 8.7	−5.69 (−7.56, −3.82)	<0.001	−4.68 (−6.89, −2.46)	<0.001	10.9 ± 2.8	−0.21 (−1.55, 1.14)	0.762	0.02 (−1.33, 1.36)	0.982
Booster-vaccinated	-	-		-		11.1 ± 2.6	−0.03 (−1.27, 1.22)	0.966	−0.01 (−1.26, 1.24)	0.986

^a^ in Delta cases, we adjusted age, gender, and whether they had comorbidities; Ct values were not adjusted since missing data for Ct-value were too large (*n* = 193, 59.2%); ^b^ in Omicron cases, we adjusted age, gender, whether they had comorbidities and Ct values at admission. β = β-coefficient. Only 4 Delta cases received booster shots and 6 Omicron cases were partially vaccinated, and these cases were excluded.

## Data Availability

The data are available from the corresponding author (doctorzzd99@163.com and 306247680@qq.com) upon reasonable request.

## Supplementary Materials

The following supporting information can be downloaded at: https://www.mdpi.com/article/10.3390/vaccines10101753/s1, Table S1: Serum Inflammation Indicators in Delta and Omicron infections ^a^. Table S2: Associations between vaccination statuses and pneumonia by subgroups with Delta infection. Table S3: Associations between vaccination statuses and pneumonia by subgroups with Omicron infection. Table S4: E-value for aRRs/β of Vaccination Statuses from the Modified Poisson Regression ^a^.
