# Supplementary material for: Effectiveness of Inactivated COVID-19 Vaccines against COVID-19 Caused by the SARS-CoV-2 Delta and Omicron Variants: A Retrospective Cohort Study
Source: Vaccines (Basel). 2022 Oct 19;10(10):1753. doi: 10.3390/vaccines10101753 (PMC9611172; doi:10.3390/vaccines10101753)
Supplement: Supplementary file 1 [file vaccines-10-01753-s001.zip › vaccines-1963130-supplementary.pdf]

Table S1 Serum Inflammation Indicators in Delta and Omicron infections[a]

Variables	Delta variant				Omicron variant			
	Unvaccinated (n = 104)	Partially vaccinated (n = 40)	Fully vaccinated (n = 182)	*P*	Unvaccinated (n=22)	Fully vaccinated (n=64)	Booster vaccination （n=139）	*P*
White blood cell counts, (×10⁹/L)	5.5 (4.4, 6.4)	5.8 (4.5, 7.2)	5.3 (4.4, 7.0)	0.576	4.9 (4.2, 5.6)	4.5 (3.7, 5.6)	5.0 (4.1, 6.4)	0.087
Neutrophil counts ($\times 10^9$/L)	3.8 (2.9, 4.9)	3.7 (3.1, 5.1)	3.5 (2.8, 4.7)	0.36	2.7 (2.1, 3.5)	2.6 (1.9, 3.6)	3.2 (2.5, 4.3)	**0.005**
Lymphocyte counts($\times 10^9$/L)	1.0 (0.7, 1.3)	1.1 (0.9, 1.4)	1.3 (1.0, 1.7)	**< 0.001**	1.5 (1.1, 2.1)	1.2 (0.9, 1.8)	1.1 (0.9, 1.5)	**0.024**
D-dimer, mg/L	0.4 (0.2, 0.5)	0.2 (0.2, 0.5)	0.3 (0.2, 0.5)	0.307	0.4 (0.2, 0.9)	0.3 (0.2, 0.5)	0.3 (0.2, 0.5)	0.235
C-reactive protein, mg/L	3.3 (3.3, 15.8)	3.3 (3.3, 11.5)	6.0 (2.0, 12.7)	0.364	1.6 (0.9, 3.4)	4.6 (1.7, 8.7)	4.1 (2.0, 8.0)	**0.021**
IL-6, pm/mL	11.8 (4.8, 30.5)	6.8 (4.8, 13.1)	3.8 (1.0, 8.7)	**< 0.001**	5.8 (4.0, 6.8)	5.5 (4.3, 6.9)	5.8 (4.7, 7.5)	0.529

[a] all laboratory findings were obtained within 48h at admission. In Delta-infected individuals, missing data for D-dimer, n=28 (8.9%); CRP, n=10 (3.1%); IL-6, n=26 (8.0%), lymphocyte counts increased and IL-6 levels decreased as vaccination doses increased. However, Omicron-infected individuals showed a decrease in lymphocyte counts and an increase in neutrophil counts and C-reactive protein

Table S2 Associations between vaccination statuses and pneumonia by subgroups with Delta infection

Subgroup	Vaccination status	Total, (n)	Event (n, %)	VE (95% CI)		P for interaction
				Unadjusted	Adjusted	
Age<60 years						0.720
	Unvaccinated	45	38 (84.4)	Reference	1(Ref)	
	Partially vaccinated	40	27 (67.5)	20% (-14%, 44%)	18% (-16%, 43%) [a]	
	Fully vaccinated	173	64 (37.0)	**56% (42%, 67%)**	56% (41%, 67%) [a]	
Age≥60 years[d]						
	Unvaccinated	59	52 (88.1)	Reference	Reference	
	Fully vaccinated	9	3 (33.3)	**62% (37%, 77%)**	64% (39%, 78%) [a]	
Without comorbidities						0.990
	Unvaccinated	52	43 (82.7)	Reference	Reference	
	Partially vaccinated	32	21 (65.6)	21% (-14%, 45%)	14% (-25%, 41%) [b]	
	Fully vaccinated	107	38 (35.5)	**57% (42%, 68%)**	**53% (34%, 66%)** [b]	
With comorbidities						
	Unvaccinated	52	47 (90.4)	Reference	Reference	
	Partially vaccinated	8	6 (75.0)	17% (-42%, 52%)	5% (-75%, 48%) [b]	
	Fully vaccinated	75	29 (38.7)	**57% (43%, 68%)**	**53% (28%, 69%)** [b]	
Male						0.218
	Unvaccinated	43	33 (76.7)	Reference	Reference	
	Partially vaccinated	20	15 (75.0)	2% (-51%, 37%)	-15% (-88%, 30%) [c]	
	Fully vaccinated	87	27 (31.0)	**60% (42%, 72%)**	**53% (28%, 69%)** [c]	
Female						
	Unvaccinated	61	57 (93.4)	Reference	Reference	
	Partially vaccinated	20	12 (60.0)	**36% (5%, 56%)**	31% (-6%, 54%) [c]	
	Fully vaccinated	95	40 (42.1)	**55% (42%, 65%)**	**51% (34%, 64%)** [c]	

[a] adjusted for gender and whether had comorbidities; [b] adjusted for gender and age; [c] adjusted for age and whether had comorbidities; [d] No patients was partially vaccinated in those greater than 60 years old.

Table S3 Associations between vaccination statuses and pneumonia by subgroups with Omicron infection

Subgroup	Vaccination status	Total, (n)	Event （n,%)	VE (95% CI)		P for interaction
				Unadjusted	Adjusted	
Age<60 years						0.640
	Unvaccinated	17	3 (17.6)	Reference	Reference	
	Fully vaccinated	56	8 (14.3)	19% (-188%, 77%)	-1% (-257%, 71% [a]	
	Booster vaccination	120	8 (6.7)	62% (-34%, 89%)	54% (-63%, 87%)[a]	
Age≥60 years						

	Unvaccinated	5	4 (80.0)	Reference		
	Fully vaccinated	8	2 (25.0)	69% (-46%，93%)	66% (-109%, 94%) [a]	
	Booster vaccination	19	2 (10.5)	**87% (38%, 9 7%)**	85% (-29%, 98%)[a]	
Without comorbidities						0.371
	Unvaccinated	16	4 (25.0)	Reference	Reference	
	Fully vaccinated	57	8 (14.0)	44% (-78%，82%)	31% (-131%, 80%)[b]	
	Booster vaccination	124	6 (4.8)	**81%(35%，94%)**	**78% (19%, 94%)** [b]	
With comorbidities						
	Unvaccinated	6	3 (50.0)	Reference	Reference	
	Fully vaccinated	7	2 (28.6)	43% (-172%, 88%)	40% (-207%, 88%) [b]	
	Booster vaccination	15	4 (26.7)	47% (-97%, 86%)	39% (-163%, 86%) [b]	
Male						0.095
	Unvaccinated	4	0 (0.0)	Reference	Reference	
	Fully vaccinated	25	5 (20.0)	-	-	
	Booster vaccination	59	5 (8.5)	-	-	
Female						
	Unvaccinated	18	7 (38.9)	Reference	Reference	
	Fully vaccinated	39	5 (12.8)	67% (2%~89%)	57% (-31%, 86%) [c]	
	Booster vaccination	80	5 (6.2)	**84% (52%, 95%)**	**75% (20%, 92%)**[c]	

[a] adjusted for gender and whether had comorbidities; [b] adjusted for gender and age; [c] adjusted for age and whether had comorbidities

Table S4 E-value for aRRs/β of Vaccination Statuses from the Modified Poisson Regression[a]

Covariate	Pneumonia	Progression to severe disease	Duration of viral shedding
**Delta infections**			
Unvaccinated	Reference	Reference	Reference
Partially vaccinated	1.530 (1.000)	1.394 (1.000)	1.619 (1.000)
Fully vaccinated	**3.586 (2.663)**	**4.567 (1.632)**	**2.647 (1.914)**
**Omicron infections**			
Unvaccinated	Reference	Reference	Reference
Fully vaccinated	2.302 (1.000)	-[b]	1.088 (1.000)
Booster vaccination	**5.702 (1.737)**	**-**	**1.063 (1.000)**

[a] Results are E-values for the aRR (E-value for the upper bound of the CI of aRR)

The E-value represents the minimum strength of association on the risk ratio scale that an unmeasured confounder would need to have with both the exposure and the outcome to fully explain away a specific exposure-outcome association, conditional on the measured covariates. E-value ranged from 2.647 to 4.567 for 2-dose aVE against pneumonia, severe COVID-19, and duration of viral shedding with Delta, and was 5.702 for 3-dose aVE against pneumonia with Omicron, indicating that a strong concurrent confounder is required to change the observed aVE. For example, the statistical significance of the VE of booster vaccination for pneumonia could be explained away if there existed an unmeasured confounder that was associated with both booster vaccination and pneumonia with a strength at least as large as an RR of 5.702. [b] No cases progressed to severe COVID-19 in Omicron cases.
